# Supplementary material for: Sedentariness of College Students Is Negatively Associated with Perceived Neighborhood Greenness at Home, but Not at University
Source: Int J Environ Res Public Health. 2019 Dec 28;17(1):235. doi: 10.3390/ijerph17010235 (PMC6982286; doi:10.3390/ijerph17010235)
Supplement: Supplementary file 1 [file ijerph-17-00235-s001.pdf]

Supplementary Materials

Sedentariness is negatively associated with Perceived Neighborhood Greenness at Home, but not at University

Alexander Karl Ferdinand Loder [1*], and Mireille Nicoline Maria van Poppel [2]

[1] Institute of Sport Science, University of Graz & Staff Department Quality Management, University of Music and Performing Arts Graz, Graz, Austria; alexlode@live.at
[2] Institute of Sport Science, University of Graz, Graz, Austria; mireille.van-poppel@uni-graz.at
* Correspondence: alexlode@live.at

Supplementary Table S1. Correlation matrix of possible confounders in the combined datasets after multiple imputation of all variables.

	**BMI**	**Physical Activity**	**Time spent sitting**	**PG home**	**PG university**	**Gender**	**Age**	**Income**	**Edu-cation**
BMI									
Physical Activity	r = -.02<br>p = .351								
Time spent sitting	r = .02<br>p = .388	**r = -.20<br>p < .001**							
Perceived Greenness at home	r = -.04<br>p = .269	r = .004<br>p = .471	r = -.09<br>p = .059						
Perceived Greenness at university	r = .02<br>p = .364	r = .01<br>p = .416	r = -.08<br>p = .082	**r = .27<br>p <.001**					
Gender	**r = .11<br>p = .026**	r = -.05<br>p = .184	r = .06<br>p = .167	r = -.02<br>p = .398	r = .01<br>p = .455				
Age	**r = .27<br>p < .001**	r = -.04<br>p = .226	r = .08<br>p = .093	r = .07<br>p = .133	r = -.03<br>p = .318	**r = .12<br>p = .024**			
Income	**r = .15<br>p =.006**	r = .02<br>p = .392	r = -.01<br>p = .452	r = .05<br>p = .175	r = -.03<br>p = .280	r = .04<br>p = .227	**r = .52<br>p < .001**		
Education	r = .05<br>p = .190	**r = -.13<br>p = .011**	r = .05<br>p = .176	r = .04<br>p = .247	r = -.07<br>p = .119	r = -.08<br>p = .086	**r = .36<br>p < .001**	**r = .22<br>p < .001**	

International Journal of
Environmental Research and Public Health

MDPI

Supplementary Table S2. Collinearity statistic of the original dataset in multivariate regression analysis for confounder testing.

**Variable**	**Tolerance**	**Variance Inflation Factor**
	BMI	
Perceived Greenness at Home	.92	1.10
Perceived Greenness at University	.92	1.09
Gender	.97	1.04
Age	.64	1.58
Income	.72	1.39
Education	.84	1.19
	Physical Activity	
Perceived Greenness at Home	.92	1.09
Perceived Greenness at University	.92	1.08
Gender	.97	1.04
Age	.65	1.53
Income	.73	1.37
Education	.85	1.18
	Sedentariness	
Perceived Greenness at Home	.91	1.10
Perceived Greenness at University	.92	1.09
Gender	.97	1.03
Age	.63	1.59
Income	.71	1.41
Education	.84	1.18

Supplementary Table S3. Linear regression models with BMI as outcome and perceived greenness as well as possible confounders as indicators (extended from Table 2).

**Analyses**	R^2	F	*df*	*b*	*t*	*df*	**95% CI *b***	*p*
				Univariate				
At home	< .001	0.002	1, 471	0.01	0.42	467	-0.02 – 0.02	.966
At university	.001	0.57	1, 467	0.01	0.75	467	-0.01 – 0.02	.449
Overall	.001	0.30	1, 471	0.01	0.56	471	-0.02 – 0.03	.579
				Multivariate				
At home and at university	.001	0.29	2, 466					.746
At home				-0.001	-0.12	466	-0.02 – 0.02	.906
At university				0.01	0.76	466	-0.01 – 0.02	.446
				Multivariate adjusted				
With confounders								
At home				-.01	-0.65	593	-0.03 – 0.01	.518
At university				.01	0.91	593	0.01 – 0.03	.361
Gender				.65	1.77	593	-0.07 – 1.38	.077
Age				.15	5.27	593	0.10 – 0.21	**< .001**
Income				-.19	-0.44	593	-1.06 – 0.67	.660
Education				-.10	-0.58	593	-0.44 – 0.24	.563

Supplementary Table S4. Linear regression models with physical activity and physical sedentariness as outcomes and perceived greenness measures as indicators (extended from Table 2).

Physical Activity (Met-h per week)								
**Analyses**	R^2	***F***	***df***	***b***	***t***	***df***	**95% CI *b***	***p***
Univariate								
At home	.002	0.90	1, 419	0.14	0.95	419	-0.15 – 0.44	.344
At university	< .001	0.01	1, 415	0.01	0.10	415	-0.23 – 0.26	.919
Overall	.001	0.38	1, 419	0.11	0.62	419	-0.24 – 0.45	.536
Multivariate								
At home and at university	.003	0.53	2, 414					.586
At home				0.16	1.03	414	-0.14 – 0.46	.304
At university				-0.02	-0.13	414	-0.27 – 0.24	.897
Multivariate adjusted								
With confounders								
At home				0.23	1.45	593	-0.08 – 0.54	.147
At university				-0.03	-0.24	593	-0.30 – 0.24	.809
Gender				-5.66	-0.93	593	-17.59 – 6.26	.352
Age				-0.28	-0.64	593	-1.13 – 0.57	.519
Income				3.52	0.56	593	-8.73 – 15.77	.573
Education				-5.59	-1.96	593	-11.18 – -0.001	.050

Sedentariness (time spent sitting, hours per day)								
**Analyses**	R^2	***F***	***df***	***b***	***t***	***df***	**95% CI *b***	***p***
				Univariate				
At home	.02	8.81	1, 471	-0.03	-2.97	471	-0.05 - -0.01	.003
At university	.01	2.39	1, 467	-0.01	-1.55	467	-0.03 – 0.003	.123
Overall	.02	7.72	1, 471	-0.03	-2.78	471	-0.05 - -0.01	.006
				Multivariate				
At home and at university	.02	4.81	2, 466					.009
At home				-0.03	-2.68	466	-0.44 - -0.01	.008
At university				-0.01	-0.83	466	-0.02 – 0.01	.532
				Multivariate adjusted				
With confounders								
At home				-0.04	-2.59	593	-0.06 – -0.01	.010
At university				-0.01	-0.70	593	-0.03 – 0.02	.487
Gender				0.22	0.54	593	-0.57 – 1.01	.587
Age				0.06	1.77	593	-0.01 – 0.12	.076
Income				-0.60	-1.31	593	-1.50 – 0.30	.192
Education				0.21	1.10	593	-0.16 – 0.58	.272

© 2019 by the authors. Submitted for possible open access publication under the terms and conditions of the Creative Commons Attribution (CC BY) license (http://creativecommons.org/licenses/by/4.0/).
